# Altered salivary microbiota associated with high-sugar beverage consumption

**DOI:** 10.1038/s41598-024-64324-w

**Published:** 2024-06-11

**Authors:** Xiaozhou Fan, Kelsey R. Monson, Brandilyn A. Peters, Jennifer M. Whittington, Caroline Y. Um, Paul E. Oberstein, Marjorie L. McCullough, Neal D. Freedman, Wen-Yi Huang, Jiyoung Ahn, Richard B. Hayes

**Affiliations:** 1 Division of Epidemiology, Department of Population Health, NYU Grossman School of Medicine, 180 Madison, New York, NY 10016 USA; 2 Laura and Isaac Perlmutter Cancer Center, NYU Langone Health, New York, NY USA; 3 Department of Epidemiology and Population Health, Albert Einstein College of Medicine, Bronx, NY USA; 4 Department of Population Science, American Cancer Society, Atlanta, GA USA; 5 Division of Cancer Epidemiology and Genetics, National Cancer Institute, Bethesda, MD USA

**Keywords:** Salivary microbiota, High-sugar beverage consumption, Oral health, Oral microbiome, Soda, Population-based study, Risk factors, Microbiome, Nutrition

## Abstract

The human oral microbiome may alter oral and systemic disease risk. Consuming high sugar content beverages (HSB) can lead to caries development by altering the microbial composition in dental plaque, but little is known regarding HSB-specific oral microbial alterations. Therefore, we conducted a large, population-based study to examine associations of HSB intake with oral microbiome diversity and composition. Using mouthwash samples of 989 individuals in two nationwide U.S. cohorts, bacterial 16S rRNA genes were amplified, sequenced, and assigned to bacterial taxa. HSB intake was quantified from food frequency questionnaires as low (< 1 serving/week), medium (1–3 servings/week), or high (> 3 servings/week). We assessed overall bacterial diversity and presence of specific taxa with respect to HSB intake in each cohort separately and combined in a meta-analysis. Consistently in the two cohorts, we found lower species richness in high HSB consumers (> 3 cans/week) (*p* = 0.027), and that overall bacterial community profiles differed from those of non-consumers (PERMANOVA *p* = 0.040). Specifically, presence of a network of commensal bacteria (*Lachnospiraceae*, *Peptostreptococcaceae,* and *Alloprevotella rava*) was less common in high compared to non-consumers, as were other species including *Campylobacter showae*, *Prevotella oulorum,* and *Mycoplasma faucium*. Presence of acidogenic bacteria *Bifodobacteriaceae* and *Lactobacillus rhamnosus* was more common in high consumers. Abundance of *Fusobacteriales* and its genus *Leptotrichia*, *Lachnoanaerobaculum sp*., and *Campylobacter* were lower with higher HSB consumption, and their abundances were correlated. No significant interaction was found for these associations with diabetic status or with microbial markers for caries (*S. mutans*) and periodontitis (*P. gingivalis*). Our results suggest that soft drink intake may alter the salivary microbiota, with consistent results across two independent cohorts. The observed perturbations of overrepresented acidogenic bacteria and underrepresented commensal bacteria in high HSB consumers may have implications for oral and systemic disease risk.

## Introduction

More than 700 bacterial species and a range of other microorganisms (archaea, fungi, and viruses) colonize the human oral cavity, known collectively as the oral microbiome^1,2^. Oral microbiota are closely tied to oral diseases, such as periodontitis and dental caries^3^, and potentially to systemic diseases, including diabetes^4,5^, cardiovascular disease^6,7^, and several types of cancer^8–11^. The salivary microbiome is increasingly preferred in studies investigating the relationship between microbiome and health, due to its non-invasive accessibility, temporal stability^12^, and its interactions with external factors and with other microbiomes (e.g. dental plaques, gut)^13,14^. However, less is known about external modifiable dietary factors associated with salivary microbiome composition.

We hypothesized that frequent consumption of high-sugar beverages (HSB), such as fruit juices and carbonated beverages, may impact the salivary microbiota. Intake of HSBs, containing high amounts of acids and fermentable carbohydrates, contributes to caries development by facilitating fermentation and selection of bacteria that thrive in a low-pH environment^15,16^. Sugars, particularly sucrose, disrupt the balance of oral microbial systems by enhancing the proportion of acid-producing bacteria and reducing alkali-producing bacteria in oral biofilms^17^. Cariogenic microorganisms in the oral microbiome not only produce more acid when metabolizing fermentable sugars^18^ but are more likely to survive in these acidic conditions compared to beneficial oral microbiota^19^. Salivary microbiota assemblages may be affected by variations in biofilm bacterial composition and the subsequently altered structure of tooth surfaces on which oral bacteria are attached. Additionally, HSB consumption elevates salivary glucose concentration, and acids found in fruit juice and carbonated beverages can decrease the pH value after consumption^20,21^, both of which impact global salivary microbiome diversity and certain bacterial phylotypes^22,23^. Finally, frequent HSB consumption may lead to inflammatory processes^24–27^ that can reshape the salivary microbiome^28^. While oral dysbiosis is likely influenced by dietary factors^29–31^, thus far there have been no examinations of the impact of HSB consumption on overall oral microbiome composition, and few studies have investigated the relationships of broader dietary patterns with the oral microbiome, with none finding significant associations^32,33^.

We tested the association of HSB consumption with the oral microbiome in 989 adults from two large U.S. cohorts, the American Cancer Society (ACS) Cancer Prevention Study-II (CPS-II) Nutrition cohort^34^ and the National Cancer Institute (NCI) Prostate, Lung, Colorectal, and Ovarian (PLCO) Cancer Screening Trial cohort^35^. The oral microbiome was characterized by bacterial 16S rRNA gene sequencing. Comparisons of overall community structure and taxonomic abundance were conducted across HSB intake groups. Sensitivity analysis was conducted to examine potential interaction or confounding effects of diabetes and specific bacterial pathogens on overall microbiome-HSB association.

## Methods

### Study population

Participants were drawn from the ACS CPS-II and NCI PLCO cohort studies. Oral wash samples were collected by mail from 70,004 CPS-II Nutrition cohort participants between 2000 and 2002 and in the PLCO control arm (n = 77,445) at recruitment from 1993 to 2001. As previously described^36^, subjects included in the present analyses were originally selected from the CPS-II and PLCO cohorts as cases (i.e. subjects who subsequently developed cancer) or controls (i.e. subjects who subsequently did not develop cancer) for collaborative nested case–control studies of the oral microbiome in relation to head and neck cancer^37^ and pancreatic cancer^9^. The study participants were all healthy at the time of sample collection. After excluding participants with implausible total energy intake (greater than 3,500 kilocalorie (kcal) or less than 500 kcal per day, n = 149) and participants without information on body mass index (BMI, n = 26), 436 participants from CPS-II (n = 157 from the head and neck study and n = 279 from the pancreas study) and 553 participants from PLCO (n = 218 from the head and neck study and n = 335 from the pancreas study) were included in the current study (Supplementary Fig. 1). All participants provided informed consent, all protocols were approved by the New York University School of Medicine Institutional Review Board (IRB), and all research was conducted in accordance with the relevant IRB guidelines and regulations.

### Assessment of HSB consumption and covariates

Dietary intake at baseline and follow-up was assessed in the PLCO cohort using a self-administered food frequency questionnaire (FFQ) that assessed usual dietary intake over the past year. This questionnaire has been validated in different studies^38–40^ and was successfully used to examine dietary intake and risk of cancer in pooled cohort studies^41–43^. We considered “orange juice or grapefruit juice,” “other 100% fruit juice or 100% fruit juice mixtures,” “other fruit drinks (such as cranberry cocktail, Hi-C, lemonade, or KoolAid, diet or regular),” and “soft drinks, soda, or pop” as HSBs for this study. CPS-II cohort participants completed a validated Willett FFQ^44–47^ in 1999 that assessed consumption of three types of regular (not sugar-free) carbonated beverages: cola-type, other caffeine-containing (e.g. Mt. Dew), and “other” (e.g. 7 Up). Participants were also asked about consumption of punch/lemonade/sugared iced tea. Frequency categories ranged from never to 4 or more servings per day. Fruit juices queried included apple juice or cider, orange juice, grapefruit juice, and other fruit juices; frequency categories ranged from never to 2 or more servings per day. The HSBs included in this study may contain a variety of sugar types, including sucrose- and fructose-based beverages, but may also include other natural and artificial sugar constituents. Comprehensive demographic and lifestyle information was collected by baseline questionnaires in both cohorts.

### Oral microbiota characterization using 16S rRNA gene amplification and sequencing

Participants in both cohorts were asked to swish vigorously with 10 mL Scope mouthwash (Proctor & Gamble; P&G, Cincinnati, OH) for 30 s, and then to expectorate into a specimen tube. This sample collection method results in similar oral microbiome profiles to that of fresh frozen unstimulated saliva, collected by allowing saliva to accumulate on the floor of the mouth^48^. Samples were shipped to each cohort’s biorepository, pelleted by centrifugation, resuspended, and aliquoted for storage at −80 °C until use^35,49^. Bacterial genomic DNA was extracted from the samples using the MoBio PowerSoil DNA Isolation Kit (Carlsbad, CA) which lyses cells for DNA extraction using mechanical (bead-based homogenization) and proprietary chemical methods that can detect both Gram-positive and Gram-negative bacteria and actinomycetes. As reported previously^50^, 16S rRNA gene sequencing on the extracted DNA was performed. 16S rRNA amplicon libraries were generated using primers incorporating FLX Titanium adapters and a sample barcode sequence, allowing unidirectional sequencing covering variable regions V3 to V4 (Primers: 347F- 5′GGAGGCAGCAGTAAGGAAT-3′ and 803R- 5′CTACCGGGGTATCTAATCC-3′). Five ng genomic DNA was used as the template in 25 μL PCR reaction buffer for 16S rRNA amplicon preparation. Cycling conditions were one cycle of 94 °C for 3 min, followed by 25 cycles of 94 °C for 15 s, 52 °C 45 s, and 72 °C for 1 min, followed by a final extension of 72 °C for 8 min. The generated amplicons were then purified using the Agencourt AMPure XP kit (Beckman Coulter, CA). Purified amplicons were quantified by fluorometry using the Quant-iT PicoGreen dsDNA Assay Kit (Invitrogen, CA). Equimolar amounts (10^7^ molecules/μL) of purified amplicons were pooled for sequencing. Pyrosequencing (Roche 454 GS FLX Titanium) was carried out according to the manufacturer’s instructions^51^.

### Upstream sequence analysis of microbiome data

16S rRNA gene amplicon sequences were processed and analyzed using the QIIME pipeline^52^. Multiplexed libraries were demultiplexed based on the barcodes assigned to each sample. Poor-quality sequences were excluded using the default parameters of the QIIME script *split_libraries.py* (minimum average quality score = 25, minimum/maximum sequence length = 200/1000 base pairs, no ambiguous base calls, and no mismatches allowed in the primer sequence). Filtered sequence reads were clustered into operational taxonomic units (OTUs) and subsequently assigned to taxa by using the Human Oral Microbiome Database (HOMD) pre-defined taxonomy map of reference sequences with ≥ 97% identity^53^. From 989 pre-diagnostic oral wash samples, we obtained 9,117,268 high-quality 16S rRNA gene sequence reads (mean 9,219 [SD 2557] sequences per sample), with a similar number of reads in all cohorts^36^. Confidence scores for the assigned taxonomic identities were calculated by QIIME using the Ribosomal Database Project (RDP) naïve Bayesian classification method^54^, with a score of 1 representing the highest possible confidence. Taxa with confidence scores ≥ 0.8 are presented in Supplementary Table 1. A summary of sequence reads per sample that were assigned to the HOMD reference is shown in Supplementary Table 2.

### Quality control

Blinded positive quality control (QC) subject specimens were used across all sequencing batches. We previously reported good agreement of microbiome parameters in replicates from these QC subjects (coefficient of variability across the four cohorts ranged from 0.45% to 8.28% for Shannon Index; 6.29% to 26% for relative abundances across various phyla)^36^. Negative control samples (with Scope mouthwash only) were used to detect possible reagent and environment contamination in all sequencing batches as well. No DNA was detected from the negative control samples. Comparing genus-level taxa detection between metataxonomics (the 16 s rRNA sequencing used in the present analysis) and metagenomics (whole-metagenome shotgun sequencing)^55^ for n = 197 ACS samples and n = 257 PLCO samples demonstrated high correlation between the two sequencing methods: Spearman correlation coefficient (mean [min–max]) for ACS cohort: (0.815 [0.319–0.983]) and PLCO cohort: (0.843 [0.228–0.998]).

### Statistical analysis

#### HSB consumption

For individuals in both cohorts, HSB consumption was defined using a standard serving size (1 serving = 12 oz or 355 g), with usual weekly consumption characterized as non-drinkers (0 servings/week), low (< 1 serving/week), medium (1–3 servings/week), or high (> 3 servings/week). To assess potential between-cohort differences in consumption, we additionally computed total HSB consumption for each cohort by summing HSB intake as grams per day from each HSB-related beverage.

#### Community composition of microbiota

Α-diversity (within-subject diversity) was assessed using species richness and the Inverse Simpson’s index, which were calculated in 500 iterations of rarefied OTU tables of 2,707 sequence reads per sample, the lowest sequencing depth among the samples. We modeled richness and Inverse Simpson’s index as outcomes in mixed-effect linear regression models accounting for the differences in HSB consumption and correlations with the outcomes across cohorts^56^, allowing the association with HSB and the predictor variables (age, sex, race, BMI, cigarette smoking status [never, former, current], alcohol consumption status [never, ever], total caloric intake [kcal], and history of diabetes) to vary across cohorts. β-diversity (between-subject diversity) was assessed by using unweighted and weighted UniFrac phylogenetic distance matrices accounting for both presence or absence of observed OTUs and their relative abundance, respectively^57^. We performed Permutational Multivariate Analysis of Variance (PERMANOVA; adonis function, vegan package, *R*)^58^ and Principal Coordinate Analysis (PCoA) to examine statistically and visually whether bacterial community composition differed by HSB intake levels. Pair-wise comparisons among intake levels for each of the first three coordinates in PCoA were conducted using the Kruskal–Wallis post hoc test (Dunn's test). PERMANOVA models considered the random effect of cohort by constraining permutations within each study stratum (CPS-II and PLCO) and all models adjusted for age, sex, race, BMI, cigarette smoking status, alcohol consumption status, total caloric intake, and history of diabetes.

#### Carriage and abundance of OTUs

OTUs were classified into 10 phyla, 24 classes, 41 orders, 72 families, 150 genera, and 496 species, according to their alignment with the HOMD reference database. In the analysis of presence/absence (carriage), we included taxa carried by at least 5% and not greater than 95% of participants, leaving 4 phyla, 11 classes, 19 orders, 35 families, 73 genera, and 271 species. Logistic regression models were used to examine the difference in carriage rate of taxa by intake levels, separately in the CPS-II and PLCO cohorts. We calculated nominal *p*-values for the CPS-II and PLCO cohorts and also report meta-analysis *p*-values based on Z-score methods^59^. In the analysis of abundance, taxa (phylum to species) with greater than 2 sequence reads in at least 100 participants were included, resulting in 8 phyla, 14 classes, 20 orders, 33 families, 55 genera, and 176 species. We used the 'DESeq' function within the DESeq2 package^60^ in *R* to fit a negative binomial generalized linear model to test for differentially abundant taxa by HSB intake level at each taxonomic level. This function models raw counts using a negative binomial distribution, which is appropriate when analyzing zero-inflated or over-dispersed counts such as microbiome taxa abundance^61^, and adjusts internally for “size factors” which normalize for differences in sequencing depth between samples. We calculated nominal *p*-values for the CPS-II and PLCO cohorts and also report meta-analysis *p*-values based on Fisher’s method^62^. In both the carriage and abundance analysis, HSB intake was treated as continuous by assigning the numbers 0, 1, 2, 3 to non-drinkers, and low-, moderate-, and high-level consumers, respectively. All models were adjusted for the above-mentioned covariates. We considered taxa with individual nominal *p*-values less than 0.10 in both cohorts and meta-*p*-values less than 0.05 as significant.

#### Sensitivity analysis

To examine if oral health status has a confounding effect on the observed HSB-microbiome associations, we additionally adjusted models for abundance of *Streptococcus mutans* (a surrogate marker for dental caries^63,64^) and carriage of *Porphyromonas gingivalis* (a surrogate marker for periodontal disease^65^). We used these surrogate markers of oral disease as we lacked information on oral health conditions in our study. We conducted several stratified analyses according to history of diabetes, smoking status, median abundance of *S. mutans,* and carriage of *P. gingivalis*. We also performed case-only analyses (separately by cohort for each cancer type) to see if the observed associations may be driven by subclinical or undetectable disease in either population. Chi-square test of Cochran’s Q statistic was used to examine heterogeneity across strata in the sensitivity analyses.

#### OTU correlation network

Spearman’s correlation of carriage rate and abundance was used to assess relationships among OTUs that were associated with HSB intake, as well as the bacterial markers for caries and periodontal disease. OTU counts were normalized for DESeq2 size factors, to account for differences in library size in a manner consistent with our differential abundance analysis, before correlation analysis. Correlation coefficients with magnitude ≥ 0.30 were selected for visualization using the “igraph” package in *R*. Partial correlation test was used to examine if the selected correlations were statistically significant after controlling for covariates, and a false discovery rate (FDR)-adjusted *p*-value < 0.05 was considered statistically significant after multiple comparison adjustment. All statistical tests were two-sided, and all statistical analyses were carried out using *R* version 3.4.0.

### Ethical approval and consent to participate

Written informed consent was obtained from all study participants, and all protocols were conducted in accordance with the U.S. Common Rule and approved by the New York University Grossman School of Medicine Institutional Review Board.

## Results

Demographic characteristics of participants by level of HSB intake are shown in Table 1. Of the 989 participants in this study, 130 (29.8%) in CPS-II and 246 (44.5%) in PLCO were non-consumers of HSBs. Participants in the highest HSB consumer group (> 3 servings/week) in CPS-II and PLCO drank, respectively, 336 and 398 g per day on average, which is greater than 1 12 oz. can of soda or juice per day. Participants were predominantly white and above middle-age. Greater HSB intake was associated with male gender, history of smoking, no history of diabetes, and higher overall caloric intake in both cohorts, and higher BMI in CPS-II only.

**Table 1:** Demographic characteristics of the study participants by levels of high-sugar beverage consumption.

		Frequency and quantity of high-sugar beverage intake				*p*-value^†^
		Non-drinker	< 1 can/week	1–3 cans/week	> 3 cans/week	
		Mean ± SD / N(%)*	Mean ± SD / N(%)	Mean ± SD / N(%)	Mean ± SD / N(%)	
**CPS-II cohort**		N = 130	N = 190	N = 57	N = 59	
High-sugar beverage intake^‡^, (g/d)		0.0 ± 0.0	20.2 ± 10.6	69.4 ± 21.5	335.7 ± 251.7	
Age		73.3 ± 6.4	73.4 ± 5.9	71.7 ± 6.1	70.7 ± 5.7	0.0028
Sex, Male		62 (47.7)	103 (54.2)	45 (78.9)	44 (74.6)	< 0.001
Race, White		129 (99.2)	186 (97.9)	55 (96.5)	56 (94.9)	0.28
BMI, kg/m^2^		26.0 ± 4.8	25.9 ± 4.5	26.4 ± 3.5	27.5 ± 4.4	0.039
Smoking status^§^						
Former		67 (51.5)	93 (48.9)	34 (59.6)	34 (57.6)	0.081
Current		6 (4.6)	4 (2.1)	3 (5.3)	9 (15.3)	< 0.001
Alcohol intake, (g/d)		8.3 ± 13.6	9.7 ± 13.7	14.5 ± 18.2	9.3 ± 12.8	0.17
Diabetes, Yes		38 (29.2)	22 (11.6)	5 (8.8)	6 (10.2)	< 0.001
Total calorie intake, (kcal/d)		1579.7 ± 466.1	1757.0 ± 495.8	2017.1 ± 672.6	2113.7 ± 533.9	< 0.001
High abundance of *S. mutans*^¶^		70 (53.8)	95 (50.0)	28 (49.1)	25 (42.4)	0.54
Carriage of *P. gingivalis*		28 (21.5)	48 (25.3)	17 (29.8)	13 (22.0)	0.63
**PLCO cohort**		N = 246	N = 162	N = 69	N = 76	
High-sugar beverage intake^‡^, (g/d)		0.0 ± 0.0	21.5 ± 12.7	91.8 ± 26.0	398.3 ± 307.7	
Age		63.8 ± 5.1	64.2 ± 5.0	63.9 ± 5.6	62.1 ± 5.3	0.056
Gender, Male		151 (61.4)	106 (65.4)	15 (78.3)	17 (77.6)	0.0092
Race, White		234 (95.1)	153 (94.4)	63 (91.3)	67 (88.2)	0.14
BMI, kg/m^2^		27.8 ± 4.7	26.3 ± 3.8	27.1 ± 3.9	27.1 ± 4.3	0.11
Smoking status^§^						
Former		125 (50.8)	62 (38.3)	24 (34.8)	26 (34.2)	0.050
Current		17 (6.9)	18 (11.1)	11 (15.9)	12 (15.8)	0.30
Alcohol intake, (g/d)		9.5 ± 13.5	10.5 ± 18.3	11.3 ± 22.3	8.9 ± 21.1	0.92
Diabetes, Yes		35 (14.2)	3 (1.9)	2 (2.9)	1 (1.3)	< 0.001
Total calorie intake, (kcal/d)		1679.6 ± 609.9	1635.2 ± 582.5	2016.4 ± 653.5	1923.2 ± 572.2	< 0.001
High abundance of *S. mutans*^¶^		124 (50.4)	71 (43.8)	37 (53.6)	45 (59.2)	0.14
Carriage of *P. gingivalis*		69 (28.0)	53 (32.7)	28 (40.6)	19 (25.0)	0.14

* Mean and standard deviation were calculated for age, BMI, alcohol intake, and caloric intake; N and percent were calculated for gender, race, smoking status, and diabetes.

^†^ *p*-values from Chi-square test or univariate linear regression model.

^‡^ High-sugar beverage intake in grams per day. 12 oz (1 can) weighs approximately 355 g.

^§^ Chi-square test was conducted for former- vs. never-smokers, and current- vs. never-smokers.

^¶^Participants with above median abundance of *S. mutans* in saliva. Cut-off was based on the normalized count of *S. mutans* in CPS-II (12.01) and in PLCO (6.95) separately.

**Figure 1:**
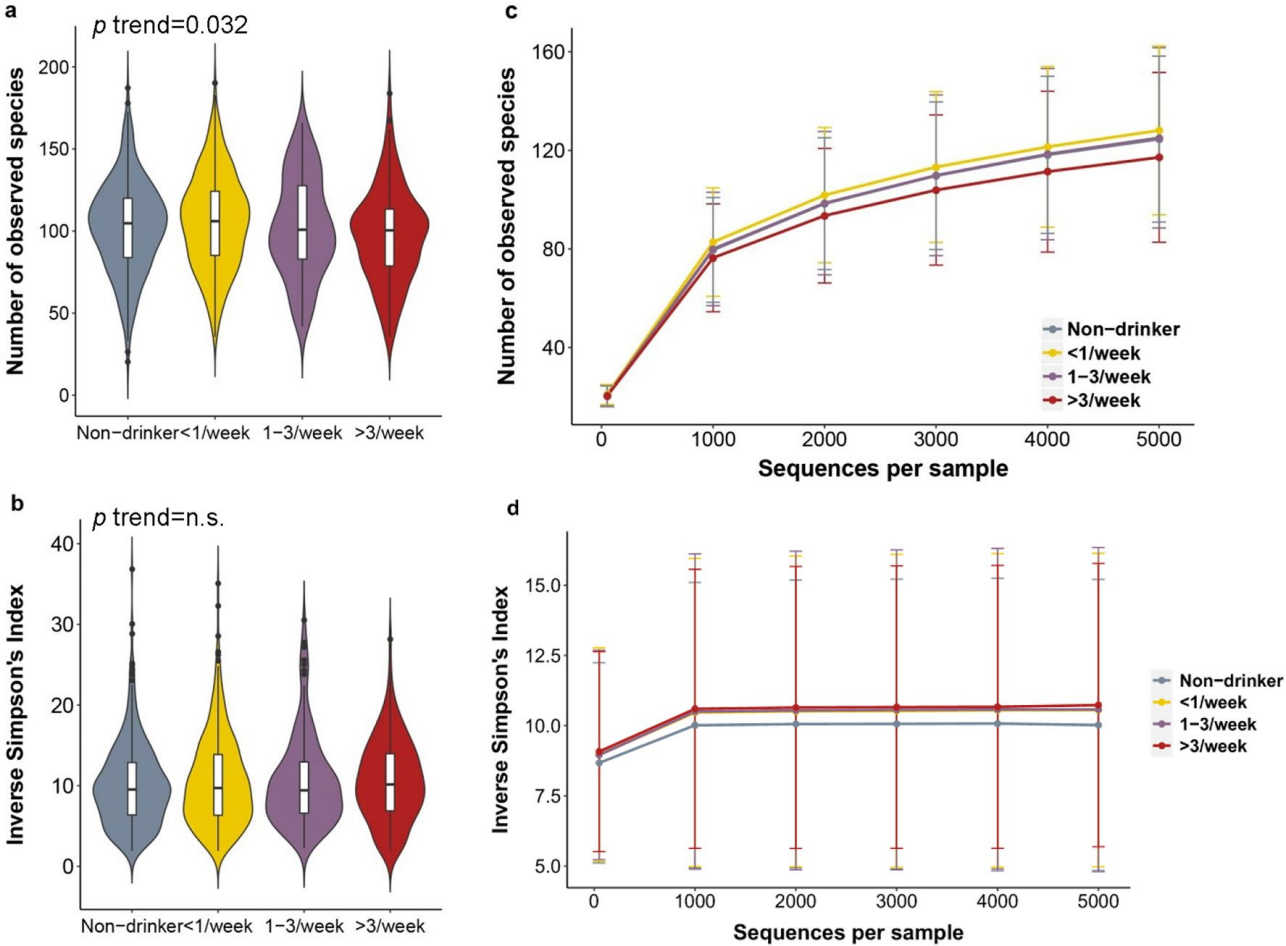
Richness and evenness of the oral microbiome by high-sugar beverage intake. (**a**,**b**) Violin plots of (**a**) number of observed OTUs (richness) and (**b**) inverse Simpson’s Index (evenness) by high-sugar beverage intake. These indices were calculated for 500 iterations of rarefied OTU tables of 2,027 sequence reads per sample, and the average over the iterations was taken for each participant. Plotted are median, interquartile ranges, and the probability density of the indices at different values. Mean values of the richness in non-drinker, low (< 1 can/week), moderate (1–3 cans/week), and high (> 3 cans/week) intake groups were 102.7, 106.0, 102.7, and 97.2; p = 0.51, 0.41, and 0.027 for each intake level compared to non-drinkers, and p = 0.032 for the trend test in linear regression model. Mean values of the inverse Simpson's Index in each group were 10.1, 10.5, 10.5, and 10.6; inverse Simpson’s index did not differ significantly by high-sugar beverage intake. (**c**,**d**) Rarefaction curves of (**c**) number of observed OTUs and (**d**) inverse Simpson’s Index according to the number of reads per sample, by high-sugar beverage intake group.

**Figure 2:**
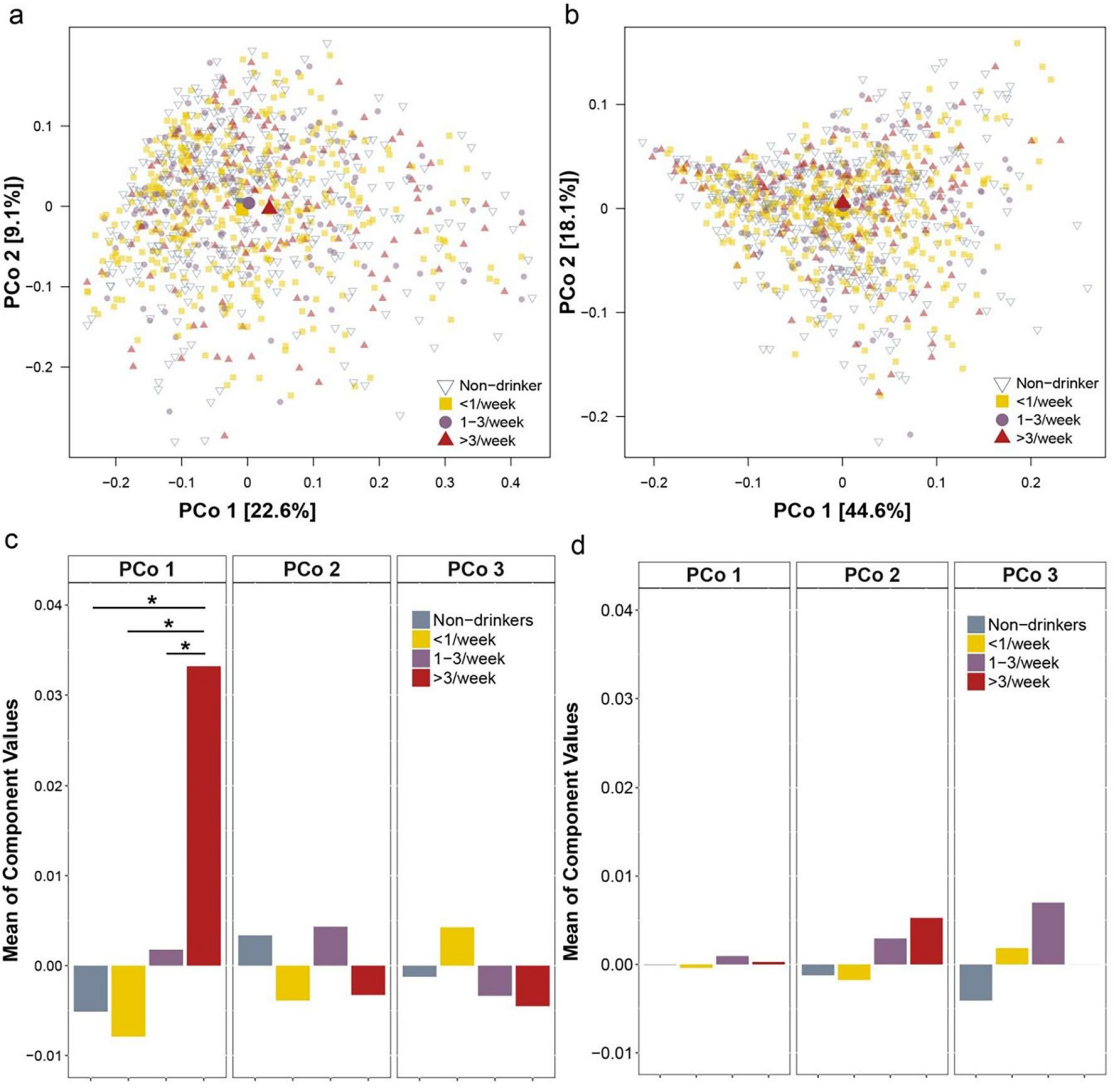
Principal Coordinate Analysis (PCoA) showing β-diversity of oral bacterial communities by high-sugar beverage intake. (a-b) PCoA plots using (**a**) unweighted and (**b**) weighted UniFrac phylogenetic distance matrices in all study participants. The community structures in non-drinkers and low- (< 1 can/week), moderate- (1–3 cans/week), and high- (> 3 cans/week) high-sugar beverage intake groups are depicted using different colors. Larger filled shapes indicate centroids for each group. (**c**,**d**) Barplots of the mean of the first three coordinates of PCoA by high-sugar beverage intake, using (**c**) unweighted and (**d**) weighted UniFrac phylogenetic distance matrices. In Adonis analysis, *p*-values for each high-sugar beverage intake group compared to non-drinkers were 0.10, 0.83, 0.44, and 0.040 using unweighted UniFrac (Kruskal–Wallis *p*-values = 0.003, 0.003, and 0.019, respectively), and 0.86, 0.18, 0.91, and 0.94 using weighted UniFrac (Kruskal–Wallis *p*-values n.s.). One star (*) indicates *p* < 0.05 in the Kruskal–Wallis post-hoc test (Dunn's test).

We first investigated how the overall community composition of oral microbiota varied according to HSB intake. When assessing α-diversity, we found that species richness decreased with higher HSB intake (Fig. 1a,c, p trend = 0.032), while evenness, measured by the inverse Simpson’s index, was not associated with HSB (Fig. 1b,d). When assessing β-diversity based on the unweighted UniFrac distance, which measures the presence or absence of bacterial lineages to define community composition, PCoA revealed that those with the highest HSB intake (Fig. 2a, large red triangle) separated from the rest on the first PCoA axis (Fig. 2a, non-drinkers, < 1/week, and 1–3/week; large gray inverted triangle, yellow square, and purple circle, respectively), and this separation was statistically significant (Fig. 2c, p = 0.003, 0.003, and 0.019 for high-, low-, and moderate-levels of intake, respectively, compared to non-drinkers, from Kruskal–Wallis post hoc test). This difference remained in PERMANOVA after controlling for covariates (*p* = 0.040 for high intake vs. non-consumer) (Supplementary Table 3). PCoA results were also similar when assessing Jaccard and Bray Curtis differences (data not shown). When assessing β-diversity based on the weighted UniFrac distance, which detects differences in relative taxa abundance, all HSB-consuming groups clustered together in PCoA (Fig. 2b), and showed no difference in the first three principal PCoA coordinates (Fig. 2d) or in PERMANOVA analysis (all *p* from post hoc test and PERMANOVA > 0.05).

**Figure 3:**
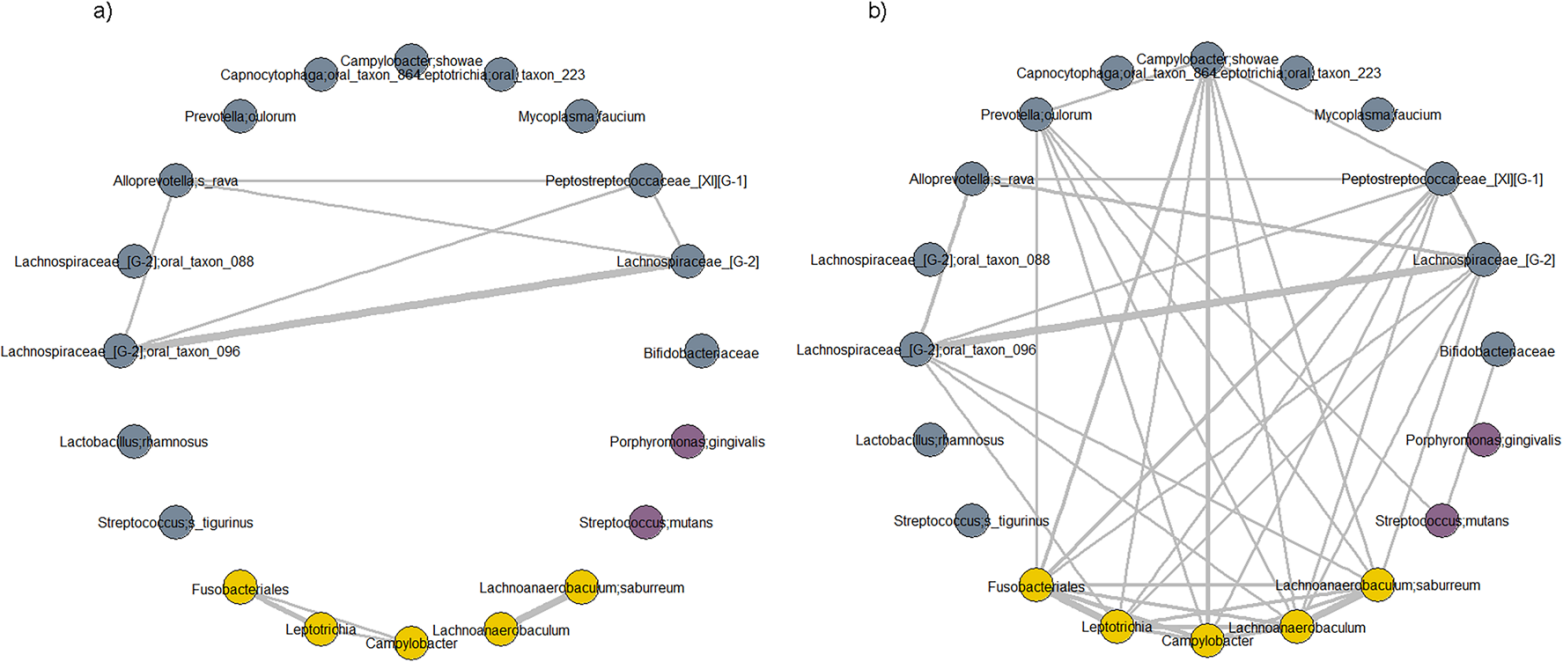
Co-occurrence and correlation networks in the oral bacterial community. (**a**,**b**) Co-occurrence and correlation network plots using (**a**) carriage rate and (**b**) abundance of taxa shown in Tables 2–3, as well as bacterial makers for caries and periodontitis. The nodes represent taxa, and edges with Spearman’s correlation coefficient |r|> 0.3 using all subjects are shown. Blue nodes represent taxa differentially carried by high-sugar beverage intake (Table 2); yellow nodes represent taxa differentially abundant by high-sugar beverage intake (Table 3); and purple nodes represent *S. mutans* and *P. gingivalis*, bacterial makers of caries and periodontitis. The thickness of edges corresponds to the coefficient values.

In the taxon-level analysis by logistic regression (Table 2), we found that higher HSB intake was associated with an increase in relative abundance of several taxa (measured as the carriage rate percentage, calculated as the number of participants who carried the taxon divided by total number of participants in each drinking level). Specifically, we observed more frequent carriage of family *Bifidobacteriaceae* (meta-*p* = 0.017) and two species: *Lactobacillus rhamnosus* (meta-*p* = 0.002) and *Streptococcus tigurinus* (meta-*p* = 0.0001). Carriage of genera *Lachnospiraceae_[G-2]* (meta-*p* = 0.003) and *Peptostreptococcaceae_[XI][G-1]* (meta-*p* = 0.001), and species *Lachnospiraceae_[G-2] sp*. (meta-*p* = 0.003) and *Alloprevotella rava* (meta-*p* = 0.008) were less abundant with higher HSB intake, and these taxa were correlated in network analysis (all FDR adjusted *p*-values from partial correlation test < 0.05, Fig. 3a). Other species with depleted carriage in HSB consumers include *Capnocytophaga sp*., *Mycoplasma faucium*, *Leptotrichia sp*., and *Campylobacter showae*.

**Table 2:** Taxa* related to high-sugar beverage intake: carriage analysis.

	Cohort	Carriage rate (%)^†^				OR^‡^	CI^‡^	*p*^‡^	Meta-*p*^§^
		Non-drinker	< 1 can /week	1–3 cans /week	> 3 cans /week				
*Actinobacteria;Actinobacteria;Bifidobacteriales;* *Bifidobacteriaceae* (F)^¶^	CPS-II	74.62	82.11	85.96	86.44	1.04	(0.99, 1.08)	0.0985	0.0171
	PLCO	79.27	82.72	78.26	88.16	1.03	(1.00, 1.06)	0.0827	
*Bacteroidetes;Bacteroidia;Bacteroidales;* *Prevotellaceae;Alloprevotella;rava* (S)	CPS-II	40.77	41.05	45.61	33.90	0.96	(0.91, 1.01)	0.0875	0.0083
	PLCO	50.00	42.59	47.83	38.16	0.96	(0.92, 1.00)	0.0439	
*Bacteroidetes;Bacteroidia;Bacteroidales;* *Prevotellaceae;Prevotella;oulorum* (S)	CPS-II	64.62	59.47	56.14	38.98	0.93	(0.88, 0.98)	0.0042	0.0003
	PLCO	55.69	55.56	42.03	40.79	0.95	(0.91, 0.99)	0.0209	
*Bacteroidetes;Flavobacteriia;Flavobacteriales;* *Flavobacteriaceae;Capnocytophaga;sp._oral_taxon_864* (S)	CPS-II	27.69	25.26	19.3	11.86	0.94	(0.90, 0.98)	0.0053	< 0.0001
	PLCO	23.98	20.99	14.49	9.21	0.95	(0.92, 0.98)	0.0020	
*Firmicutes;Bacilli;Lactobacillales;* *Lactobacillaceae;Lactobacillus;rhamnosus* (S)	CPS-II	8.46	13.68	8.77	20.34	1.03	(0.99, 1.07)	0.0977	0.0018
	PLCO	8.94	9.26	10.14	18.42	1.04	(1.01, 1.06)	0.0078	
*Firmicutes;Bacilli;Lactobacillales;* *Streptococcaceae;Streptococcus;tigurinus* (S)	CPS-II	5.38	4.74	3.51	15.25	1.04	(1.02, 1.07)	0.0016	0.0001
	PLCO	3.66	1.85	17.39	7.89	1.02	(1.01, 1.04)	0.0111	
*Firmicutes;Clostridia;Clostridiales;* *Lachnospiraceae_[XIV];Lachnospiraceae_[G-2]* (G)	CPS-II	65.38	63.68	64.91	49.15	0.94	(0.89, 0.99)	0.0161	0.0029
	PLCO	69.11	64.81	53.62	63.16	0.96	(0.93, 1.00)	0.0586	
*Firmicutes;Clostridia;Clostridiales;* *Lachnospiraceae_[XIV];Lachnospiraceae_[G-2];sp._oral_taxon_088* (S)	CPS-II	11.54	6.32	8.77	3.39	0.97	(0.95, 1.00)	0.0653	0.0070
	PLCO	14.63	8.64	11.59	5.26	0.97	(0.95, 1.00)	0.0473	
*Firmicutes;Clostridia;Clostridiales;* *Lachnospiraceae_[XIV];Lachnospiraceae_[G-2];sp._oral_taxon_096* (S)	CPS-II	63.08	62.63	61.40	47.46	0.94	(0.89, 0.99)	0.0166	0.0026
	PLCO	66.67	61.73	50.72	60.53	0.96	(0.92, 1.00)	0.0514	
*Firmicutes;Clostridia;Clostridiales;* *Peptostreptococcaceae_[XI];Peptostreptococcaceae_[XI][G-1]* (G)	CPS-II	73.08	73.68	71.93	64.41	0.96	(0.91, 1.00)	0.0763	0.0010
	PLCO	79.27	73.46	65.22	65.79	0.95	(0.92, 0.98)	0.0050	
*Firmicutes;Mollicutes;Mycoplasmatales;* *Mycoplasmataceae;Mycoplasma;faucium* (S)	CPS-II	13.85	10.00	10.53	6.78	0.97	(0.94, 1.00)	0.0522	0.0086
	PLCO	12.60	6.79	5.80	7.89	0.98	(0.95, 1.00)	0.0693	
*Fusobacteria;Fusobacteriia;Fusobacteriales;* *Leptotrichiaceae;Leptotrichia;sp._oral_taxon_223* (S)	CPS-II	20.77	20.53	10.53	8.47	0.96	(0.92, 1.00)	0.0321	0.0085
	PLCO	11.79	11.73	11.59	3.95	0.98	(0.95, 1.00)	0.0964	
*Proteobacteria;Epsilonproteobacteria;Campylobacterales;* *Campylobacteraceae;Campylobacter;showae* (S)	CPS-II	75.38	71.58	71.93	61.02	0.94	(0.90, 0.99)	0.0213	0.0048
	PLCO	69.92	69.14	72.46	53.95	0.97	(0.93, 1.00)	0.0759	

*Taxa were selected if *p*-value < 0.10 detected in logistic regression in both cohorts, and meta-*p*-value < 0.05 performed using Z-score method.

^†^Carriage rate was the number of participants who carried the taxon divided by total number of participants in each drinking level.

^‡^Odds ratios and *p*-values are calculated based on logistic regression models controlled for age, race [White, non-White], sex, BMI category, smoking status [never-, former-, and current smokers], alcohol consumption status [never- and ever drinkers], grams of ethanol per day, history of diabetes, and total caloric intake. High-sugar beverage intake level was treated as a continuous variable by assigning the numbers 0, 1, 2, 3 to non-drinkers and each level of intake, respectively.

^§^Meta-analysis *p-*values from logistic regression models within each of the 2 cohorts, calculated using Z-score methods.

^¶^Association was significant up to order *Bifidobacteriales*, because this family is the single constituent member of its order.

In the linear analysis of oral taxa abundance according to levels of HSB intake (Table 3), we found that order *Fusobacteriales* (meta-*p* = 0.038) and its major genus *Leptotrichia* (meta-*p* = 0.025), as well as *Lachnoanaerobaculum* (meta-*p* = 0.01) and its species *L. saburreum* (meta-*p* = 0.001), and *Campylobacter* (meta-*p* = 0.025) were less abundant with higher HSB intake. Network analysis showed that abundances of these taxa were also highly correlated (all FDR adjusted *p*-values from partial correlation test < 0.05, Fig. 3b).

**Table 3:** Taxa* related to high-sugar beverage intake: abundance analysis.

	Cohort	Carriage rate (%)^†^				OR^‡^	CI^‡^	*p*^‡^	Meta-*p*^§^
		Non-drinker	< 1 can /week	1–3 cans /week	> 3 cans /week				
*Firmicutes;Clostridia;Clostridiales;* *Lachnospiraceae_[XIV];Lachnoanaerobaculum* (G)	CPS-II	4.14	4.15	6.65	2.05	0.84	(0.75, 0.95)	0.0056	0.0096
	PLCO	2.72	2.53	2.06	2.23	0.88	(0.82, 0.96)	0.0026	
*Firmicutes;Clostridia;Clostridiales;* *Lachnospiraceae_[XIV];Lachnoanaerobaculum;saburreum* (S)	CPS-II	3.54	4.16	5.26	1.64	0.82	(0.71, 0.94)	0.0059	0.0006
	PLCO	2.07	1.78	2.00	2.10	0.79	(0.70, 0.89)	0.0001	
*Fusobacteria;Fusobacteriia;Fusobacteriales* (O)^¶^	CPS-II	343.75	371.88	299.07	318.46	0.90	(0.83, 0.97)	0.0066	0.0380
	PLCO	145.64	169.62	180.30	163.28	0.95	(0.89, 1.00)	0.0658	
*Fusobacteria;Fusobacteriia;Fusobacteriales;* *Leptotrichiaceae;Leptotrichia* (G)^#^	CPS-II	130.15	120.27	83.03	87.99	0.85	(0.76, 0.94)	0.0015	0.0252
	PLCO	61.07	67.76	60.51	67.94	0.93	(0.86, 1.00)	0.0592	
*Proteobacteria;Epsilonproteobacteria;Campylobacterales;* *Campylobacteraceae;Campylobacter* (G)**	CPS-II	37.40	45.82	34.56	30.04	0.88	(0.81, 0.97)	0.0078	0.0252
	PLCO	24.91	21.38	20.92	20.53	0.92	(0.86, 0.99)	0.0196	

*Taxa were selected if *p-*value < 0.10 detected by DESeq function in both cohorts, and meta-*p*-value < 0.05 performed using the Fisher method.

^†^Sequence read counts were normalized by dividing raw counts by DESeq size factors**.**

^‡^ The association between taxonomic abundance and high-sugar beverage intake level was detected by DESeq function, adjusted for age, race [White, non-White], sex, BMI category, smoking status [never-, former-, and current smokers], alcohol consumption status [never- and ever drinkers], grams of ethanol per day, history of diabetes, and total caloric intake. Nominal *p-*values from trend tests. In trend test, high-sugar beverage intake level was treated as a continuous variable by assigning the numbers 0, 1, 2, and 3 to non-drinker and each level of intake, respectively.

^§^Meta-*p*-value combination using Fisher method performed with “fishercomb” function in metaRNASeq *R* package.

^¶^Association was significant up to class *Fusobacteriia*, because this order is the single constituent member of its class.

^#^Association was significant up to family *Leptotrichiaceae*, because this genus is the single constituent member of its family.

** Association was significant up to class *Epsilonproteobacteria*, because this genus is the single constituent member of its class.

As diabetes and the oral diseases of dental caries and periodontitis are related to both HSB use and oral microbiome composition, we examined whether the observed associations of the oral microbiome and HSB intake were influenced by diabetes status (yes/no) and by oral bacterial markers for dental caries (*S. mutans*) and periodontitis (*P. gingivalis*) (Supplementary Tables 4–5). The finding of lower richness with higher HSB intake remained after further adjustment for abundance of *S. mutans* and carriage of *P. gingivalis* (p = 0.024 and 0.017, respectively) and history of diabetes (*p* = 0.090). No evidence of heterogeneity across strata was observed in stratified analyses for history of diabetes, smoking status, and markers of dental caries or periodontitis, and magnitude of the associations were attenuated in the case-only analyses. In taxon-based analysis, further adjustment for abundance of *S. mutans* and carriage of *P. gingivalis* did not affect the results shown in Tables 2, 3 (data not shown).

## Discussion

In this study we observed, for the first time, that greater HSB intake is associated with lower bacterial richness and altered bacterial composition in the saliva. Some acidogenic bacteria were overrepresented in those with higher HSB intake, while certain commensal bacteria were significantly lower. These findings were consistent in two independent cohorts. Additionally, these associations were independent of history of diabetes and microbial markers of caries and periodontitis, suggesting that the associations are not mediated by diabetic and oral health conditions. The strength of the associations decreased in the case-only analyses, suggesting that the findings are not driven by underlying cancer risk factors.

We observed that greater HSB intake was associated with decreased richness of the salivary microbiota. A more diverse bacterial community typically results in higher stability and an enhanced capacity to respond to changes in the environment, while decreased diversity is often associated with disease states^5,66–70^. The lower richness we observed with HSB intake may result from a direct impact of HSBs on the oral microbiota, or simply reflect the poor oral health conditions in HSB consumers. Poor oral health conditions, including higher plaque index, presence of decayed teeth, and deeper periodontal pockets could be related to both HSB consumption and salivary microbial diversity^71–73^. However, sensitivity analyses using surrogate bacterial markers of oral disease showed no significant confounding effect.

We also identified certain phylotypes overrepresented in those with higher HSB intake. The increase of *Bifidobacteriaceae* and *L. rhamnosus*, taxa with high acidogenic capacity^64,74–76^, may result from ecological changes in the mouth due to HSB consumption. In addition, a member of the non-mutans *S. mitis* group, *S. tigurinus*, was increased in consumers. This species was recently isolated from a periodontitis patient^77^ and is a suspected component of pathogenic biofilms^78^. In contrast, the abundance of *S. mutans*, which is specifically linked to dental caries^64^, was not related to HSB intake in our study. This suggests that other dietary factors, including other sources of dietary sugars and carbohydrates, may play a larger role in promoting *S. mutans* growth^64^. This result is also consistent with the current evidence that, while salivary *S. mutans* is strongly associated with caries prevalence^63^, other bacteria able to produce substantial amounts of acid from fermentable carbohydrates may also contribute to caries development^79^.

Several commensal bacteria were underrepresented in those with higher HSB intake. Commensal bacteria play important roles in the homeostasis of microbiome and host, and their depletion may disturb the innate immunity of gingival tissue and lead to subsequent health conditions^80^. For example, butyric acid-producing *Lachnospiraceae* is important for both microbial and host epithelial cell growth^81^, and oral *Fusobacteria* and its major genus *Leptotrichia* are associated with decreased risk of pancreatic cancer^9^. We also identified two positive co-occurrence and correlation networks among some of the HSB-related phylotypes. The underlying mechanisms for this may include nutritional cross-feeding, co-aggregation, co-colonization, signaling pathways, and co-survival in similar environments^82–85^. Though confirmation of these bacterial networks is needed, our results provide some insights into potential probiotics for oral health, particularly targeting those with high HSB intake. *Lactobacilli* and *Bifidobacteria* are common intestinal probiotics and have been considered as potential probiotics for oral health as well, due to negligible pathogenicity, lack of toxic fermentation products, production of antimicrobial compounds, and stimulation of immunity^86^. However, the acidogenic properties of *Lactobacilli* and *Bifidobacteria* can lead to caries^87–90^, highlighting the complex bacterial interactions which may be responsible for oral disease. In this study, higher HSB intake was related to greater carriage of *L. rhamnosus* and *Bifidobacteriaceae*; these taxa were unrelated to other HSB-depleted commensal phylotypes, while the abundance of *Bifodobacteriaceae* was positively correlated with *S. mutans* in network analysis. These findings indicate that *Lactobacilli* and *Bifidobacteria* may not be good candidates for oral system probiotics^91^.

Our study has several strengths. First, the use of 16S rRNA gene sequencing for microbiome analysis allowed us to comprehensively assess overall oral bacterial community composition and specific taxon abundances. Second, our very large sample size provided excellent statistical power to detect variation in the oral microbiome with respect to HSB intake in two independent cohorts and allowed us to confirm our findings across sub-groups of interest. Finally, the detailed demographic and lifestyle information allowed us to adjust for potential confounding factors. A limitation of our study is that it is observational and cross-sectional, limiting the ability to establish a causal relationship. A longitudinal trial where those with moderate or high HSB intake were randomized to continue or stop HSB consumption could provide more definitive information. Further, the majority of study participants were White and above middle-age, limiting the generalizability of our findings to other races and age groups. Because the samples were collected by the participants outside of a controlled clinical environment, we do not have information on the specific conditions under which they were collected, such as the food or medications consumed that day or what time of day they were collected. We also lacked information on the oral health status of the study participants, though sensitivity analysis using bacterial markers as proxies of dental caries (dental decay) and periodontitis (gingival disease) suggest that the observed HSB-microbiota associations are independent of oral diseases. Using bacterial surrogates of two distinct oral health indicators affecting different tissues provides orthogonal evidence supporting this claim^92^. Given the evolving landscape of multi-omics technologies, future analyses using more advanced sequencing techniques are needed to validate these findings. In particular, confirming the species-level results using greater sequencing depth and increased sequence homology with the HOMD reference database (e.g. ≥ 98.5%), will be important, as not all species in our analysis met the 0.8 confidence threshold for taxa assignment. Lastly, 16S rRNA gene sequencing data alone limits our ability to understand the functional activities of the salivary microbial community related to HSB intake.

## Conclusions

We found that HSB intake is related to overall salivary microbiome community composition and the abundance of specific oral taxa. Greater HSB intake was associated with greater prevalence of acidogenic bacteria and depletion of commensal bacteria. Such changes may potentially contribute to HSB-related diseases, including caries, periodontitis, oral cancer, and diabetes. The taxa we have identified can be further investigated to elucidate their potential role in HSB-related health conditions. Future studies should also investigate the impact of HSB intake on the metagenomic (functional) content of the oral microbiome. Improved understanding of the causes and health impacts of HSB consumption and oral microbiome composition can lead to diet and microbiome-targeted approaches for disease prevention, such as HSB substitution and probiotics for oral health.

### Supplementary Information

Supplementary Information 1.

Supplementary Information 2.

## Data Availability

The datasets analyzed during the current study are available in the dbGaP repository, dbGaP Study Accession: phs001286.v1.p1 https://www.ncbi.nlm.nih.gov/projects/gap/cgi-bin/study.cgi?study_id=phs001286.v1.p1.

## Abbreviations

ACS: American Cancer Society
CPS-II: Cancer Prevention Study-II
FDR: False discovery rate
FFQ: Food frequency questionnaire
HSB: High-sugar beverage
HOMD: Human Oral Microbiome Database
NCI: National Cancer Institute
OTU: Operational taxonomic unit
PCR: Polymerase chain reaction
PERMANOVA: Permutational analysis of variance
PLCO: Prostate, Lung, Colorectal, and Ovarian Cancer Screening Trial
PCoA: Principal coordinate analysis
QC: Quality control
