# Supplementary material for: Altered salivary microbiota associated with high-sugar beverage consumption
Source: Sci Rep. 2024 Jun 11;14:13386. doi: 10.1038/s41598-024-64324-w (PMC11167035; doi:10.1038/s41598-024-64324-w)
Supplement: Supplementary file 1 — Supplementary Information 1. [file 41598_2024_64324_MOESM1_ESM.pdf]

Altered Salivary Microbiota Associated with High-Sugar Beverage Consumption

Authors and Affiliations: Xiaozhou Fan[1]*, Kelsey R. Monson[1,2]*, Brandilyn A. Peters[1,3], Jennifer M. Whittington[2], Caroline Y. Um[4], Paul E. Oberstein[2], Marjorie L. McCullough[4], Neal D. Freedman[5], Wen-Yi Huang[5], Jiyoung Ahn[1,2], Richard B. Hayes[1,2]

[1]Division of Epidemiology, Department of Population Health, NYU Grossman School of Medicine, New York, New York.
[2]Laura and Isaac Perlmutter Cancer Center, NYU Langone Health, New York, New York.
[3]Department of Epidemiology and Population Health, Albert Einstein College of Medicine, Bronx, New York
[4]Department of Population Science, American Cancer Society, Atlanta, Georgia.
[5]Division of Cancer Epidemiology and Genetics, National Cancer Institute, Bethesda, Maryland.

* These authors contributed equally to this work

Supplementary Figure 1: Population flow diagram illustrating study exclusion criteria and final study population for analysis. NCI PLCO: National Cancer Institute Prostate, Lung, Colorectal, and Ovarian Cancer Screening Trial; ACS CPS-II: American Cancer Society Cancer Prevention Study II; H&N: Head and Neck cancer

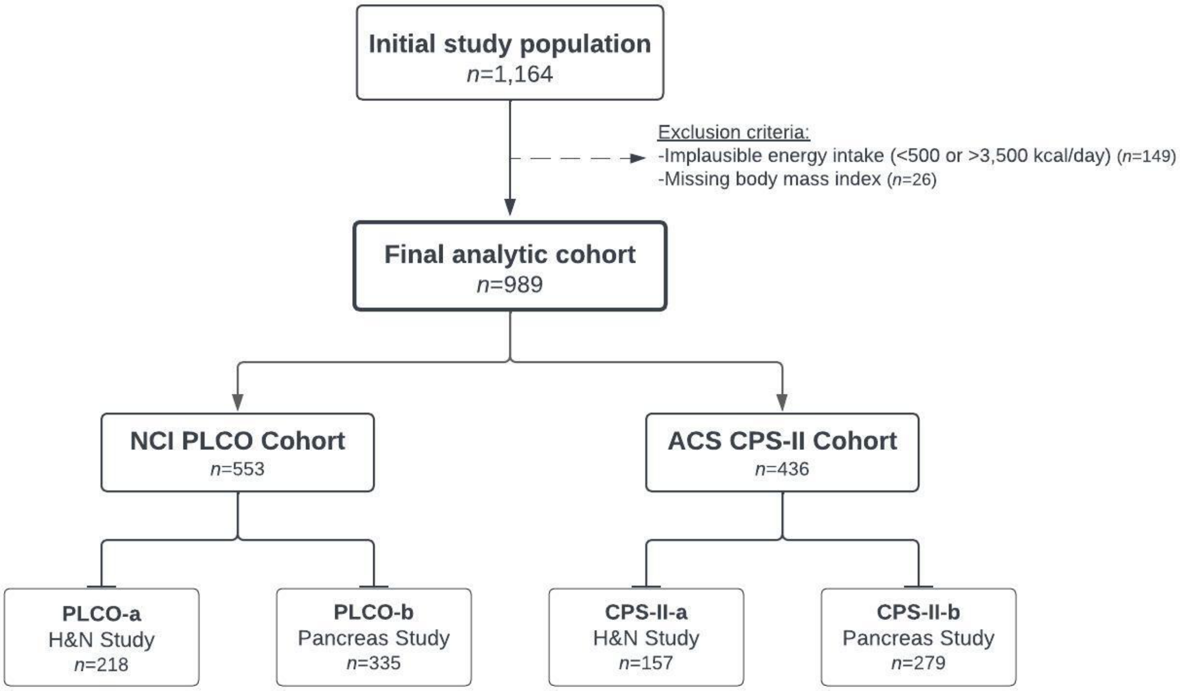

Supplementary Table 2: Number of sequence reads per sample* assigned to reference taxonomy map.

Cohort†	Minimum	Maximum	Mean	SD
CPSII-a	5,290	24,248	8,982	2,998
CPSII-b	2,865	16,132	8,799	2,072
PLCO-a	2,707	27,039	7,812	2,862
PLCO-b	4,731	23,765	10,595	1,707
Total	2,707	27,039	9,219	2,557

* Sequence reads per sample which were assigned to taxa by using the Human Oral Microbiome Database (HOMD) pre-defined taxonomy map of reference sequences with ≥97% identity.

† CPSII-a and CPSII-b indicate the head and neck study and pancreas study within CPS-II cohort, respectively; PLCO-a and PLCO-b indicate the head and neck study and pancreas study within PLCO cohort.

Supplementary Table 3: Association of distance β-diversity metrics with High Sugar Beverage intake, PLCO and CPS-II cohorts

	P-value[a]				
	**Non-drinker**	**<1 can/week**	**1-3 cans/week**	**>3 cans/week**	***P*-trend**
**HSB Intake**	*Ref.*	0.825	0.441	0.040	0.140

[a]*P*-values are from PERMANOVA of unweighted UniFrac distance adjusted for age, sex, current smoking, history of diabetes, BMI (kg/m2), energy intake (kcal/day), alcohol consumption (yes/no), and alcohol intake (grams/day). Permutations were constrained within each study stratum (PLCO and CPS-II) to account for the random effect of cohort.

Supplementary Table 4: Taxa* carriage rate related to high-sugar beverage intake level stratified by (a) history of diabetes, (b) abundance of *S. mutans*, and (c) carriage of *P. gingivalis* in the CPS-II and PLCO cohorts.

a) History of Diabetes		CPS-II			PLCO		
	Strata	OR†	CI†	*p*†	OR†	CI†	*p*†
Bifidobacteriaceae	Yes	1.04	(0.93, 1.16)	0.4794	1.03	(0.86, 1.24)	0.7129
	No	1.02	(0.98, 1.07)	0.3311	1.03	(0.99, 1.06)	0.1092
Alloprevotella;rava	Yes	0.95	(0.84, 1.09)	0.4830	0.78	(0.58, 1.05)	0.1074
	No	0.96	(0.90, 1.02)	0.1459	0.96	(0.92, 1.01)	0.0892
Prevotella;oulorum	Yes	0.97	(0.85,1.11)	0.6974	0.88	(0.66, 1.17)	0.3875
	No	0.91	(0.86, 0.97)	0.0025	0.95	(0.92, 1.00)	0.0334
Capnocytophaga;sp._oral_taxon_864	Yes	0.96	(0.88, 1.05)	0.3854	0.98	(0.78, 1.23)	0.8620
	No	0.93	(0.88, 0.98)	0.0068	0.95	(0.92, 0.98)	0.0017
Lactobacillus;rhamnosus	Yes	1.14	(1.05, 1.23)	0.0016	0.94	(0.77, 1.15)	0.5278
	No	1.01	(0.97, 1.05)	0.7945	1.03	(1.01, 1.06)	0.0086
Streptococcus;tigurinus	Yes	1.01	(0.94, 1.09)	0.7380	1.09	(1.00, 1.19)	0.0644
	No	1.04	(1.02, 1.07)	0.0025	1.02	(1.00, 1.04)	0.0185
Lachnospiraceae_[G-2]	Yes	0.90	(0.80, 1.01)	0.0900	0.99	(0.77, 1.26)	0.9137
	No	0.94	(0.89, 0.99)	0.0299	0.97	(0.93, 1.01)	0.0882
Lachnospiraceae_[G-2];sp._oral_taxon_088	Yes	0.98	(0.93, 1.04)	0.5603	0.89	(0.70, 1.14)	0.3708
	No	0.97	(0.94, 1.00)	0.0630	0.98	(0.95, 1.00)	0.0709
Lachnospiraceae_[G-2];sp._oral_taxon_096	Yes	0.90	(0.80, 1.01)	0.0900	1.03	(0.80, 1.32)	0.8217*
	No	0.94	(0.88, 0.99)	0.0293	0.96	(0.92, 1.00)	0.0702*
Peptostreptococcaceae_[XI][G-1]	Yes	0.99	(0.87, 1.12)	0.8240	1.11	(0.91, 1.35)	0.3065
	No	0.95	(0.90, 1.00)	0.0438	0.95	(0.91, 0.98)	0.0056
Mycoplasma;faucium	Yes	0.97	(0.89, 1.00)	0.5374	0.94	(0.78, 1.13)	0.5007
	No	0.96	(0.93, 1.00)	0.0411	0.98	(0.96, 1.00)	0.0895
Leptotrichia;sp._oral_taxon_223	Yes	0.95	(0.87, 1.03)	0.2325	1.01	(0.81, 1.27)	0.8992
	No	0.96	(0.92, 1.01)	0.0867	0.98	(0.95, 1.00)	0.0974
Campylobacter;showae	Yes	0.98	(0.86, 1.11)	0.7133	0.92	(0.73, 1.15)	0.4573
	No	0.93	(0.88, 0.98)	0.0068	0.97	(0.93, 1.01)	0.1324

b) Abundance of *S. mutans*[‡]		CPS-II			PLCO		
	**Strata**	**OR**[†]	**CI**[†]	***p***[†]	**OR**[†]	**CI**[†]	***p***[†]
Bifidobacteriaceae	Low	1.04	(0.97, 1.11)	0.2740	1.02	(0.97, 1.08)	0.4614
	High	1.04	(0.99, 1.10)	0.0850	1.02	(0.98, 1.06)	0.2789
Alloprevotella;rava	Low	0.98	(0.91, 1.05)	0.5679	0.95	(0.89, 1.01)	0.0941
	High	0.93	(0.86, 1.00)	0.0482	0.95	(0.90, 1.01)	0.1135
Prevotella;oulorum	Low	0.97	(0.90, 1.05)	0.4808	0.92	(0.86, 0.97)	0.0049
	High	0.88	(0.82, 0.95)	0.0009	0.95	(0.90, 1.01)	0.1062
Capnocytophaga;sp._oral_taxon_864	Low	0.96	(0.90, 1.02)	0.1793	0.92	(0.88, 0.97)	0.0014
	High	0.91	(0.85, 0.98)	0.0087	0.97	(0.92, 1.01)	0.1712
Lactobacillus;rhamnosus	Low	0.99	(0.96, 1.02)	0.4163*	1.01	(0.99, 1.04)	0.3406
	High	1.08	(1.01, 1.14)	0.0234*	1.05	(1.01, 1.10)	0.0240
Streptococcus;tigurinus	Low	1.04	(1.00, 1.09)	0.0389	1.00	(0.98, 1.03)	0.7935*
	High	1.04	(1.01, 1.07)	0.0112	1.05	(1.02, 1.07)	0.0008*
Lachnospiraceae_[G-2]	Low	0.95	(0.89, 1.03)	0.2024	0.91	(0.86, 0.96)	0.0007
	High	0.93	(0.86, 1.00)	0.0470	1.01	(0.96, 1.07)	0.6127
Lachnospiraceae_[G-2];sp._oral_taxon_088	Low	0.97	(0.93, 1.01)	0.1220	0.97	(0.93, 1.01)	0.1080
	High	0.98	(0.94, 1.02)	0.2842	0.98	(0.95, 1.02)	0.2816
Lachnospiraceae_[G-2];sp._oral_taxon_096	Low	0.96	(0.89, 1.03)	0.2386	0.91	(0.86, 0.96)	0.0010*
	High	0.93	(0.86, 0.99)	0.0363	1.01	(0.96, 1.07)	0.6680*
Peptostreptococcaceae_[XI][G-1]	Low	0.98	(0.93, 1.04)	0.6018	0.94	(0.89, 0.99)	0.0120
	High	0.93	(0.86, 1.00)	0.0526	0.96	(0.91, 1.01)	0.1146
Mycoplasma;faucium	Low	0.97	(0.92, 1.01)	0.1323	0.97	(0.93, 1.00)	0.0508
	High	0.97	(0.92, 1.02)	0.2429	0.98	(0.95, 1.02)	0.3525
Leptotrichia;sp._oral_taxon_223	Low	0.99	(0.93, 1.05)	0.7629	0.99	(0.96, 1.03)	0.6998
	High	0.92	(0.87, 0.98)	0.0075	0.96	(0.92, 0.99)	0.0176
Campylobacter;showae	Low	0.91	(0.86, 0.97)	0.0047	0.92	(0.87, 0.98)	0.0069
	High	0.97	(0.90, 1.04)	0.4156	1.00	(0.95, 1.06)	0.9757

c) **Carriage of *P. gingivalis***		**CPS-II**			**PLCO**		
	**Strata**	**OR**†	**CI**†	***p***†	**OR**†	**CI**†	***p***†
Bifidobacteriaceae	Yes	1.01	(0.92, 1.10)	0.8978	1.01	(0.96, 1.07)	0.6518
	No	1.04	(0.99, 1.09)	0.0854	1.03	(0.99, 1.07)	0.1517
Alloprevotella;rava	Yes	0.98	(0.87, 1.10)	0.7265	0.98	(0.90, 1.07)	0.6395
	No	0.95	(0.90, 1.01)	0.0972	0.94	(0.89, 0.98)	0.0068
Prevotella;oulorum	Yes	0.88	(0.78, 0.98)	0.0243	0.98	(0.90, 1.07)	0.6547
	No	0.94	(0.89, 1.00)	0.0444	0.94	(0.90, 0.99)	0.0210
Capnocytophaga;sp._oral_taxon_864	Yes	0.96	(0.87, 1.06)	0.4695	0.91	(0.84, 0.98)	0.0093
	No	0.94	(0.89, 0.98)	0.0092	0.96	(0.92, 1.00)	0.0303
Lactobacillus;rhamnosus	Yes	1.02	(0.96, 1.09)	0.5358	1.03	(0.98, 1.09)	0.2001
	No	1.03	(0.99, 1.07)	0.1572	1.04	(1.01, 1.07)	0.0084
Streptococcus;tigurinus	Yes	1.06	(1.00, 1.11)	0.0450	1.01	(0.97, 1.04)	0.7354
	No	1.04	(1.01, 1.07)	0.0156	1.03	(1.01, 1.06)	0.0101
Lachnospiraceae_[G-2]	Yes	1.02	(0.92, 1.13)	0.7076	0.99	(0.92, 1.06)	0.7929
	No	0.92	(0.87, 0.98)	0.0055	0.94	(0.90, 0.99)	0.0177
Lachnospiraceae_[G-2];sp._oral_taxon_088	Yes	1.00	(0.94, 1.07)	0.9893	0.99	(0.93, 1.05)	0.7763
	No	0.97	(0.94, 1.00)	0.0317	0.96	(0.94, 0.99)	0.0145
Lachnospiraceae_[G-2];sp._oral_taxon_096	Yes	1.01	(0.91, 1.12)	0.8181	0.99	(0.92, 1.07)	0.8393
	No	0.92	(0.87, 0.98)	0.0084	0.94	(0.90, 0.99)	0.0204
Peptostreptococcaceae_[XI][G-1]	Yes	0.97	(0.87, 1.07)	0.5355	0.96	(0.90, 1.02)	0.1929
	No	0.96	(0.91, 1.01)	0.1039	0.94	(0.90, 0.98)	0.0065
Mycoplasma;faucium	Yes	0.90	(0.81, 0.99)	0.0331	0.94	(0.88, 1.00)	0.0640
	No	0.99	(0.96, 1.02)	0.4058	0.98	(0.96, 1.01)	0.1333
Leptotrichia;sp._oral_taxon_223	Yes	0.95	(0.87, 1.03)	0.2142	0.98	(0.93, 1.04)	0.5033
	No	0.96	(0.92, 1.01)	0.0817	0.98	(0.95, 1.01)	0.1199
Campylobacter;showae	Yes	0.98	(0.89, 1.07)	0.5896	0.98	(0.92, 1.05)	0.5766
	No	0.94	(0.89, 0.99)	0.0229	0.96	(0.91, 1.01)	0.0805

All *p* values from heterogeneity test were < 0.05 using the Chi-square test of Cochran's Q statistic.

* Taxa from main text Table 2.

† Odds ratios and p values are calculated based on logistic regression models controlled for age, race [White, non-white], sex, BMI category, smoking status [never-, former-, and current smokers], alcohol consumption status [never- and ever drinkers], grams of ethanol per day, and total caloric intake. History of diabetes was further adjusted in b) and c). High-sugar beverage intake level was treated as a continuous variable by assigning the numbers 0, 1, 2, and 3 to non-drinker and each level of intake group, respectively.

‡ Participants in each drinking level stratified by *S. mutans* abundance. Cut-off was based on the median normalized count of *S. mutans* in CPS-II (12.01) and in PLCO (6.95) separately.

Supplementary Table 5: Taxa* abundance related to high-sugar beverage intake level stratified by (a) history of diabetes, (b) abundance of *S. mutans*, and (c) carriage of *P. gingivalis* in the CPS-II and PLCO cohorts.

a) History of diabetes		CPS-II			PLCO		
	Strata	OR†	CI†	*p*†	OR†	CI†	*p*†
Lachnoanaerobaculum (G)	Yes	0.90	(0.69, 1.18)	0.4460	0.77	(0.46, 1.31)	0.3373
	No	0.84	(0.74, 0.95)	0.0060	0.89	(0.82, 0.96)	0.0034
Lachnoanaerobaculum;saburreum (S)	Yes	1.16	(0.84, 1.61)	0.3696*	0.58	(0.31, 1.08)	0.0842
	No	0.77	(0.65, 0.90)	0.0011*	0.79	(0.69, 0.89)	0.0002
Fusobacteriales (O)	Yes	0.90	(0.72, 1.14)	0.3887	0.63	(0.39, 1.04)	0.0722
	No	0.83	(0.74, 0.92)	0.0007	0.94	(0.87, 1.01)	0.0969
Leptotrichia (G)	Yes	1.03	(0.84, 1.26)	0.7590	0.82	(0.59, 1.13)	0.2200
	No	0.86	(0.79, 0.94)	0.0005	0.95	(0.90, 1.01)	0.0818
Campylobacter (G)	Yes	0.98	(0.80, 1.21)	0.8683	0.85	(0.53, 1.37)	0.5073
	No	0.86	(0.78, 0.95)	0.0023	0.92	(0.86, 0.99)	0.0256
**b) Abundance of *S. mutans*‡**							
Lachnoanaerobaculum (G)	Yes	0.89	(0.79, 0.99)	0.0297	0.90	(0.77, 1.05)	0.1744
	No	0.88	(0.74, 1.04)	0.1261	0.83	(0.75, 0.92)	0.0007
Lachnoanaerobaculum;saburreum (S)	Yes	0.78	(0.65, 0.93)	0.0075	0.81	(0.68, 0.98)	0.0287
	No	0.90	(0.74, 1.09)	0.2889	0.74	(0.64, 0.85)	0.0000
Fusobacteriales (O)	Yes	0.94	(0.87, 1.02)	0.1272	0.88	(0.79, 0.99)	0.0261
	No	0.83	(0.74, 0.93)	0.0017	0.97	(0.90, 1.05)	0.4910
Leptotrichia (G)	Yes	0.92	(0.83, 1.02)	0.1061	0.89	(0.78, 1.01)	0.0797
	No	0.81	(0.70, 0.94)	0.0073	0.94	(0.86, 1.04)	0.2525
Campylobacter (G)	Yes	0.91	(0.83, 1.01)	0.0764	0.83	(0.73, 0.94)	0.0040
	No	0.86	(0.76, 0.98)	0.0241	0.94	(0.86, 1.04)	0.2325
**c) Carriage of *P. gingivalis***							
Lachnoanaerobaculum (G)	Yes	0.89	(0.75, 1.05)	0.1755	0.94	(0.81, 1.10)	0.4502
	No	0.86	(0.75, 0.99)	0.0367	0.83	(0.73, 0.93)	0.0018
Lachnoanaerobaculum;saburreum (S)	Yes	0.75	(0.56, 1.01)	0.0546	0.90	(0.75, 1.08)	0.2663
	No	0.86	(0.73, 1.02)	0.0803	0.75	(0.65, 0.88)	0.0002
Fusobacteriales (O)	Yes	0.96	(0.83, 1.10)	0.5530	0.93	(0.83, 1.05)	0.2486
	No	0.89	(0.82, 0.97)	0.0087	0.95	(0.89, 1.03)	0.2148
Leptotrichia (G)	Yes	0.87	(0.75, 1.03)	0.0992	0.78	(0.68, 0.90)	0.0005
	No	0.86	(0.76, 0.97)	0.0118	1.02	(0.92, 1.12)	0.7620
Campylobacter (G)	Yes	0.92	(0.79, 1.07)	0.2684	0.89	(0.78, 1.02)	0.0974
	No	0.89	(0.80, 0.99)	0.0287	0.91	(0.83, 1.01)	0.0730

All *p* from heterogeneity test were < 0.05 using the Chi-square test of Cochran's Q statistic.

* Taxa from main text Table 3.

† The association between taxonomic abundance and high-sugar beverage intake level was detected by DESeq function, adjusted for age, race [White, non-white], sex, BMI category, smoking status [never-, former-, and current smokers], alcohol consumption status [never- and ever drinkers], grams of ethanol per day, and total caloric intake. History of diabetes was further adjusted in b) and c). Nominal *p* values from trend tests. In trend test, high-sugar beverage intake level was treated as a continuous variable by assigning the numbers 0, 1, 2, 3 to non-drinkers and each group of intake, respectively.

‡ Participants in each drinking level stratified by *S. mutans* abundance. Cut-off was based on the median normalized count of *S. mutans* in CPS-II (12.01) and in PLCO (6.95) separately.
